# Transcriptomic Profiling Identifies DCBLD2 as a Diagnostic and Prognostic Biomarker in Pancreatic Ductal Adenocarcinoma

**DOI:** 10.3389/fmolb.2021.659168

**Published:** 2021-03-23

**Authors:** Zengyu Feng, Kexian Li, Yulian Wu, Chenghong Peng

**Affiliations:** ^1^Department of General Surgery, Pancreatic Disease Center, Ruijin Hospital, Shanghai Jiao Tong University School of Medicine, Shanghai, China; ^2^Research Institute of Pancreatic Diseases, Shanghai Jiao Tong University School of Medicine, Shanghai, China; ^3^Department of General Surgery, The Second Affiliated Hospital, School of Medicine, Zhejiang University, Hangzhou, China

**Keywords:** DCBLD2, pancreatic ductal adenocarcinoma, prognosis, diagnosis, extracellular vesicles, immune infiltrates

## Abstract

Background: Accumulating evidence shows that the elevated expression of DCBLD2 (discoidin, CUB and LCCL domain-containing protein 2) is associated with unfavorable prognosis of various cancers. However, the correlation of DCBLD2 expression value with the diagnosis and prognosis of pancreatic ductal adenocarcinoma (PDAC) has not yet been elucidated. Methods: Univariate Cox regression analysis was used to screen robust survival-related genes. Expression pattern of selected genes was investigated in PDAC tissues and normal tissues from multiple cohorts. Kaplan–Meier (K–M) survival curves, ROC curves and calibration curves were employed to assess prognostic performance. The relationship between DCBLD2 expression and immune cell infiltrates was conducted by CIBERSORT software. Biological processes and KEGG pathway enrichment analyses were adopted to clarify the potential function of DCBLD2 in PDAC. Results: Univariate analysis, K–M survival curves and calibration curves indicated that DCBLD2 was a robust prognostic factor for PDAC with cross-cohort compatibility. Upregulation of DCBLD2 was observed in dissected PDAC tissues as well as extracellular vesicles from both plasma and serum samples of PDAC patients. Both DCBLD2 expression in tissue and extracellular vesicles had significant diagnostic value. Besides, DCBLD2 expression was correlated with infiltrating level of CD8^+^ T cells and macrophage M2 cells. Functional enrichment revealed that DCBLD2 might be involved in cell motility, angiogenesis, and cancer-associated pathways. Conclusion: Our study systematically analyzed the potential diagnostic, prognostic and therapeutic value of DCBLD2 in PDAC. All the findings indicated that DCBLD2 might play a considerably oncogenic role in PDAC with diagnostic, prognostic and therapeutic potential. These preliminary results of bioinformatics analyses need to be further validated in more prospective studies.

## Introduction

Pancreatic ductal adenocarcinoma (PDAC) is one of the deadliest cancers in the world with a 5-year survival rate lower than 9% (Siegel et al., 2020). Curative resection is the only established treatment and it remarkably improves the 5-year survival rate to 20–30% (Kamisawa et al., 2016). Unfortunately, only minority of PDAC patients have surgical indication since over 80% patients are diagnosed with unresectable, advanced-stage tumors (Wong and Raman, 2010). Accumulating evidences have demonstrated that diagnosis of PDAC at an earlier, resectable stage is likely to result in dramatic improvement of patient outcome (Ideno et al., 2020). Thus, novel diagnostic and prognostic biomarkers of PDAC with satisfactory sensitivity and specificity is urgently needed.

The discoidin, CUB and LCCL domain-containing protein 2 (DCBLD2) is a type-I transmembrane protein which has been discovered to have considerable tumor-specific functions (Schmoker et al., 2019). DCBLD2 is upregulated in lung cancer and promotes cell motility (Koshikawa et al., 2002). In colorectal cancer, high DCBLD2 expression is associated with poor patient survival, as well as tumorigenesis, invasion and metastasis of cancer cells (He et al., 2020). In gastric cancer, DCBLD2 is downregulated by epigenetic modification, and it exhibits a suppressive role in cancer cell proliferation and invasion (Kim et al., 2008). In the context of PDAC, the elevated expression of DCBLD2 is correlated with poor clinical outcome (Raman et al., 2018; Feng et al., 2020). However, the prognostic value of DCBLD2 was evaluated solely in microarray data (Feng et al., 2020), which does not capture mRNA expression as accurately as RNA-Sequencing (Zhao et al., 2014). Compared with clinical predictors such as age and histological grade, the superiority of DCBLD2 expression for survival prediction is largely unclarified. In addition, although DCBLD2 was frequently reported to be upregulated in PDAC, its diagnostic value is rarely studied, especially in extracellular vesicles.

In this study, we aim to integrate transcriptome data and survival data from multiple cohorts and platforms in order to comprehensively and systematically analysis the diagnostic and prognostic potential of DCBLD2 in PDAC. The prognostic performance was validated in both microarray data and RNA-sequencing data to ensure compatibility and reliability. The diagnostic value was investigated in both PDAC tissues and extracellular vesicles from serum and plasma samples. The considerable clinical relevance of DCBLD2 may facilitate early detection and personalized treatment of PDAC.

## Materials and Methods

### PDAC Cohorts

The ten PDAC cohorts included in this study for survival analyses were the MTAB-6134 cohort (*N* = 288), PACA-AU cohort (*N* = 62), PACA-CA cohort (*N* = 181), TCGA cohort (*N* = 139), and six microarray cohorts, GSE21501 (*N* = 97), GSE28735 (*N* = 42), GSE57495 (*N* = 63), GSE62452 (*N* = 64), GSE71729 (*N* = 123), and GSE85916 (*N* = 79). Eleven cohorts including GSE15471, GSE16515, GSE28735, GSE32676, GSE41368, GSE55643, GSE60979, GSE62165, GSE62452, GSE71729, and GSE71989 were employed to evaluate the expression of genes. All these cohorts contained both PDAC tissue samples and normal tissue samples. In addition, GSE133684 cohort, which provided expression profiles of extracellular vesicles in human plasma samples from PDAC, chronic pancreatitis (CP) and healthy individuals, was chosen to investigate the potential implication of genes in liquid biopsy. The normalized gene expression data and clinical information of all GSE cohorts were downloaded from the Gene Expression Omnibus^1^ (GEO). Data of MTAB-6134 cohort was downloaded from ArrayExpress database^2^. All data of PACA-AU and PACA-CA cohorts was obtained from the International Cancer Genome Consortium^3^ (ICGC). The TCGA data was obtained from the TCGA hub at UCSC Xena^4^. In each cohort, patients with incomplete clinical data or with a histopathological type other than PDAC were removed from this study. Patients with a survival time of <1 month were excluded. The baseline characteristics of all PDAC patients with global clinical information are detailed in Supplementary Table S1.

### In-House Serum Samples

Serum samples from patients with CP (*n* = 5), PDAC (*n* = 73), and from healthy donors (*n* = 42) were collected at the Department of General Surgery of Ruijin Hospital from March 2018 to December 2018 and frozen at −80°C. None of the patients received preoperative chemotherapy or radiotherapy. Written informed consent was obtained from all patients. The Ethics Committee of Ruijin Hospital affiliated with Shanghai Jiao Tong University approved the study (No. 121 in 2017).

### Survival-Related Genes Screening

The univariate Cox regression analysis was conducted to identify the survival-related genes in six independent microarray cohorts (GSE21501, GSE28735, GSE57495, GSE62452, GSE71729, and GSE85916 cohorts). Venn diagram^5^ was used to screen common prognostic genes with *P* < 0.05 in all six cohorts. The identified genes were deemed as robust survival-related genes.

### Prognostic Validation of Robust Survival-Related Genes

Patients in each cohort were divided into low- and high-expression groups according to the optimal cut-off value calculated by X-Tile software (Camp et al., 2004). Kaplan–Meier (K–M) survival curves were utilized to assess the survival differences between low- and high-expression groups. Calibration plots comparing the predicted and observed clinical outcome were adopted to evaluate the predictive performance. ROC curves were used to compare the efficiency of genes with that of clinical indicators for prognosis prediction.

### Estimation of Tumor Immune Infiltrates

We estimated the relative proportions of the 22 subtype immune cells in each sample by the software CIBERSORT. Samples with a *P* < 0.05 were included. The CIBERSORT software can use the deconvolution algorithm to estimate the composition of immune infiltrating cells according to gene expression matrix (Chen et al., 2018). We further used the Pearson correlation analyses to determine the correlation of gene expression level with the immune infiltrates.

### Functional Enrichment Analysis

To shed light on the biological function, co-expressed genes (*P* < 0.05) were screened by Pearson correlation analysis in MTAB-6134 cohort and TCGA cohort respectively. Top 1,000 positively correlated genes were subjected to biological process and The Kyoto Encyclopedia of Genes and Genomes (KEGG) pathway enrichment analysis on DAVID online website (Huang et al., 2009).

### Isolation of Extracellular Vesicles and Extracellular Vesicular RNA

For each case, 1.2 mL of serum was used, and an exoRNeasy Serum/Plasma Kit (Qiagen, Hilden, Germany) was used to extract extracellular vesicles following the manufacturer’s instructions. Extracellular vesicles were eluted with 100 μL phosphate-buffered saline (PBS), and half of them were used for characterization and the rest for RNA isolation. Morphology of extracellular vesicles was observed by transmission electron microscopy (TEM, JEOL, Japan) on a JEOL-1230 instrument. The density and size distribution of the extracellular vesicles were measured by nanoparticle tracking analysis (NTA) using a ZetaView PMX 110 (Particle Metrix, Germany). Extracellular vesicular RNA was extracted using QIAzol (Qiagen, Hilden, Germany).

### Quantitative Real-Time Polymerase Chain Reaction (qRT-PCR)

The extracellular vesicular RNAs of total 120 samples (Ruijin cohort) were reverse-transcribed using an Evo M-MLV RT Kit (Accurate Biology, China). Real-time PCR was conducted with an ABI 7900 instrument using ChamQ SYBR qPCR Master Mix (Vazyme, Nanjing, China). Quantitation was performed in triplicate and expression was computed using the 2^–Δ^ ^Δ^ ^*CT*^ method. Glyceraldehyde-3-phosphate dehydrogenase (GAPDH) was used as an internal reference. The primer sequences for amplified mRNAs are as follows: DCBLD2-Forward: 5′-GCTCCAACTCCTCCTCCTTCTCC-3′; DCBLD2-Reverse: 5′-GTGTCCACATCCATCACCTTGCTG-3′; GPADH-Forward: 5′- GCACCGTCAAGGCTGAGAAC-3′; GAPDH-Reverse: 5′- TGGTGAAGACGCCAGTGGA-3′.

### Statistical Analysis

The statistical analysis and graphical work were carried out in the R environment (version 3.5.2). Cox regression analyses and K–M survival curves were plotted by the “survival” package. The ROC curves for diagnosis were derived from the “pROC” package while the ROC curves for prognosis were generated from the “survivalROC” package. Boxplots were depicted using the “ggpubr” package. Calibration curves were produced by the “rms” package. Correlation curves were plotted by the “ggstatsplot” package. The enriched pathways and biological processes were illustrated by the “ggplot2” package. A two-sided log-rank *P* < 0.05 was considered significant.

## Results

### DCBLD2 Was Associated With Unfavorable Survival in PDAC

**FIGURE 1:**
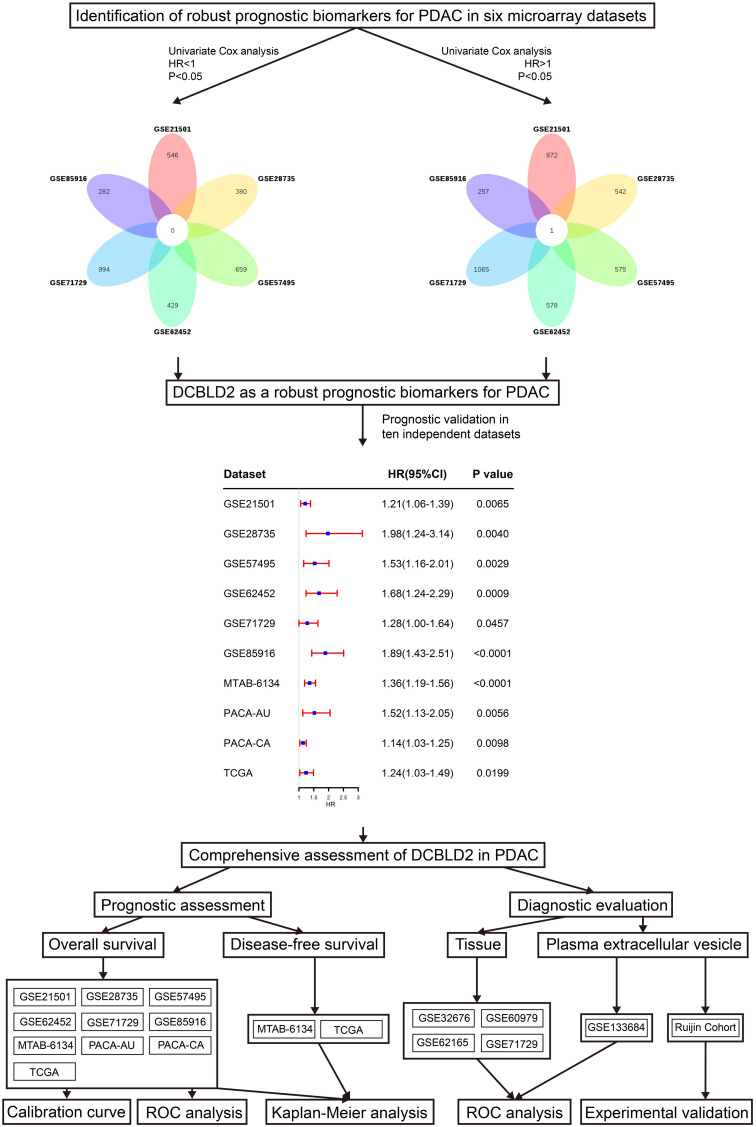
Overall flowchart study design and data analyses of this study.

Figure 1 shows the research workflow of this study. Univariate Cox regression analysis was first applied to screen survival-related genes and hundreds of prognostic genes in each cohort were identified. Subsequently Venn diagram revealed that only DCBLD2 was consistently correlated with patient survival in six independent microarray cohorts. We then evaluated the prognostic value of DCBLD2 in four RNA-sequencing cohorts. The results showed that in all these abovementioned cohorts, DCBLD2 was significantly associated with overall survival (OS) of patients (GSE21501: HR = 1.21, 95% CI = 1.06–1.39, *P* = 0.0065; GSE28735: HR = 1.98, 95% CI = 1.24–3.14, *P* = 0.004; GSE57495: HR = 1.53, 95% CI = 1.16–2.01, *P* = 0.0029; GSE62452: HR = 1.68, 95% CI = 1.24–2.29, *P* = 0.0009; GSE71729: HR = 1.28, 95% CI = 1.00–1.64, *P* = 0.0457; GSE85916: HR = 1.89, 95% CI = 1.43–2.51, *P* < 0.0001; MTAB-6134: HR = 1.39, 95% CI = 1.19–1.56, *P* < 0.0001; PACA-AU: HR = 1.52, 95% CI = 1.13–2.05, *P* = 0.0056; PACA-CA: HR = 1.14, 95% CI = 1.03–1.25, *P* = 0.0098; TCGA: HR = 1.24, 95% CI = 1.03–1.49, *P* = 0.0199).

### DCBLD2 Is Upregulated in PDAC With Diagnostic Potential

**FIGURE 2:**
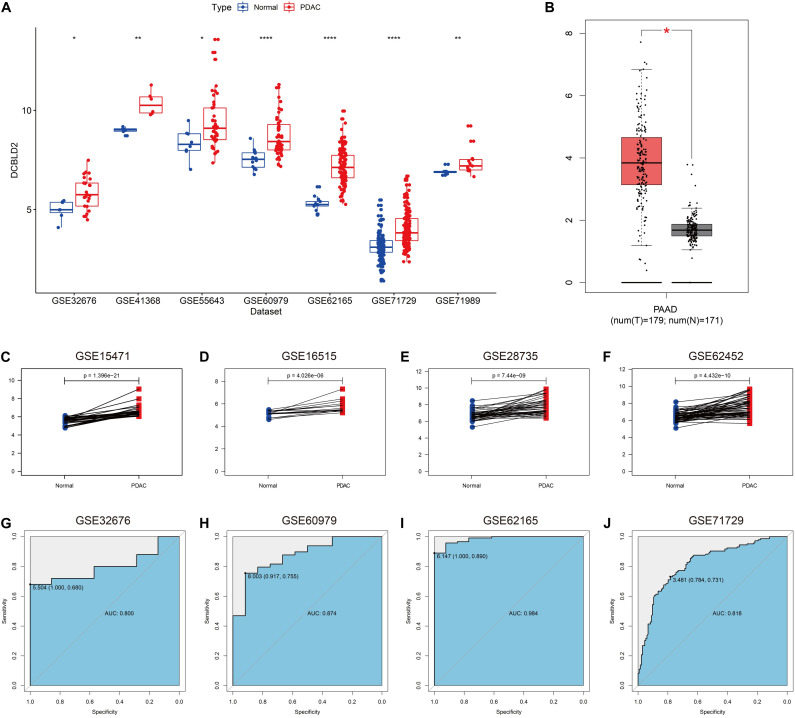
Expression of DCBLD2 in PDAC tissues and its diagnostic value. **(A)** The expression of DCBLD2 in unpaired PDAC tissues and normal tissues in seven independent PDAC cohorts. **(B)** Expression profile of DCBLD2 based on the GEPIA database. **(C–F)** Expression pattern of DCBLD2 in PDAC tissues and matched adjacent normal tissues in four independent PDAC cohorts. **(G–J)** ROC curves illustrated the value of DCBLD2 in the diagnosis of PDAC in four independent PDAC cohorts. The statistical significance of differential expression was assessed by Wilcoxon test (*p < 0.05, **p < 0.01 and ****p < 0.0001).

As illustrated in Figure 2A, DCBLD2 was remarkably overexpressed in PDAC tissues compared with unpaired normal tissues in seven independent GEO cohorts. According to the Gene Expression Profiling Interactive Analysis (GEPIA) database (Tang et al., 2017), DCBLD2 expression was significantly elevated in PDAC tissues (Figure 2B). Figures 2C–F demonstrated that DCBLD2 expression was also significantly increased in PDAC tissues compared with paired normal tissues. Furthermore, we explored the diagnostic potential of DCBLD2 in four GEO cohorts (GSE32676, GSE60979, GSE62165, and GSE71729). The area under the curve (AUC) values of DCBLD2 for diagnosis were 0.800, 0.874, 0.984, and 0.818, respectively (Figures 2G–J). This finding suggested that diagnostic accuracy of DCBLD2 was comparable to that of an established diagnostic marker, CA19-9, whose AUC value was approximately 0.84 (Xing et al., 2018). In addition, the expression of DCBLD2 was markedly increased in patients with high grade (*P* < 0.05), suggesting that DCBLD2 was related to high tumor malignancy (Supplementary Figure 1).

### Prognostic Performance of DCBLD2

**FIGURE 3:**
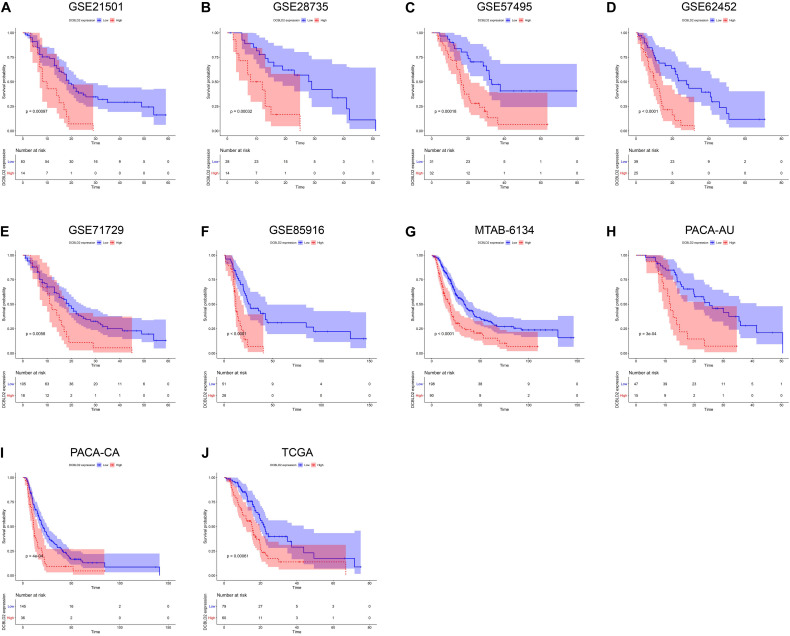
Prognostic validation of DCBLD2 in PDAC. **(A–J)** K–M curves estimated the OS difference between low- and high-expression groups in ten independent PDAC cohorts. The statistical significance of differential survival was evaluated by log-rank test.

**FIGURE 4:**
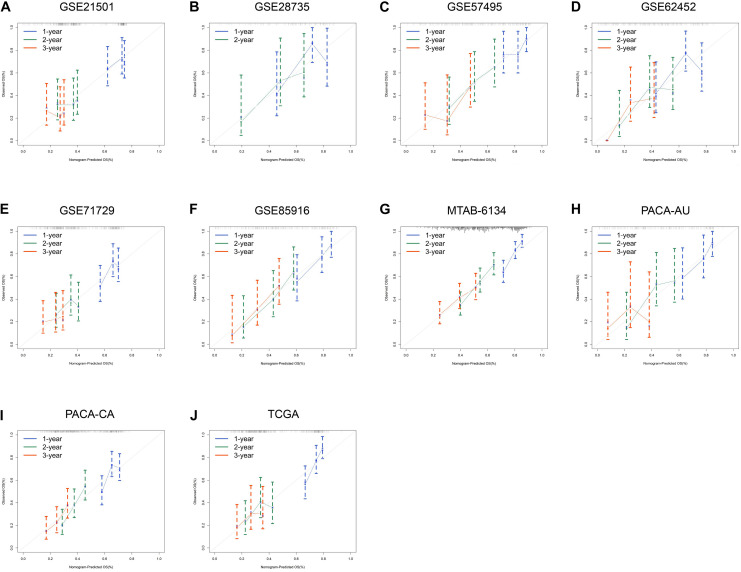
Prognostic performance of DCBLD2 in PDAC. **(A–J)** Calibration curves for DCBLD2 in ten independent PDAC cohorts.

We next assessed the prognostic efficiency of DCBLD2 in 10 independent PDAC cohorts. K–M survival curves illustrated that patients in the high-expression group of DCBLD2 had a significant shorter OS than patients in the low-expression group (Figures 3A–J). The calibration curves revealed that the predicted survival probabilities by DCBLD2 were in good accordance with the observed survival probabilities (Figures 4A–J).

**FIGURE 5:**
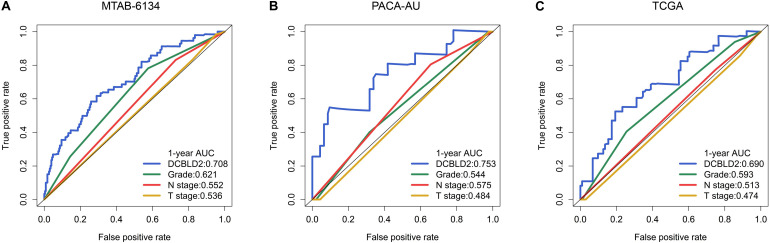
Comparison of predictive accuracy of DCBLD2 and clinical parameters. **(A–C)** ROC curves compared the predictive abilities of DCBLD2 and clinical parameters for OS in the MTAB-6134, PACA-AU, and TCGA cohorts, respectively.

We further compared the robustness of DCBLD2 with clinical indicators, including histological grade, N stage and T stage, in MTAB-6134, PACA-AU, and TCGA cohorts. The AUC values of DCBLD2 for 1-year OS prediction were 0.708, 0.753, and 0.690, respectively, which were higher than those of clinical factors in all three cohorts (Figures 5A–C). This finding suggested that DCBLD2 outperformed traditional indicators in predicting PDAC survival. Moreover, patients in the high-expression group had a significantly decreased disease-free survival (DFS) compared with patients in the low-expression group in MTAB-6134 and TCGA cohorts, indicating that DCBLD2 may also serve as a prognostic indicator of DFS (Supplementary Figure 2).

### Relationship Between Immune Cell Infiltration and DCBLD2 Expression

**FIGURE 6:**
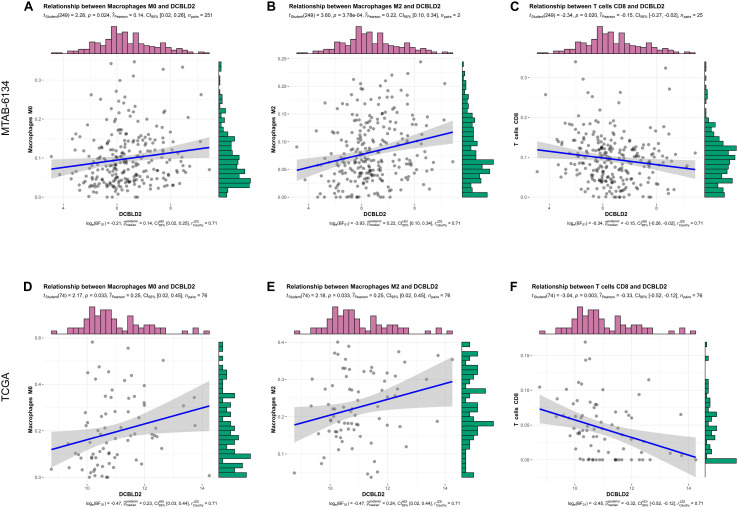
Correlation analysis between DCBLD2 expression and immune infiltrates. **(A–C)** The correlation between DCBLD2 expression and the abundance of macrophage M0, macrophage M2, and CD8^+^ T cell in MTAB-6134 cohort, respectively. **(D–F)** The correlation between DCBLD2 expression and the infiltration of macrophage M0, macrophage M2 and CD8^+^ T cell in TCGA cohort, respectively. The correlation coefficients and *p* value were derived from Pearson correlation analysis.

The infiltration level of immune cells is strongly associated with the clinical effects of immunotherapy and the prognosis of PDAC patients (Inman et al., 2014). Consequently, we investigated the relationship between DCBLD2 expression and immune cell infiltration and explored the possibility of DCBLD2 as the precise predictor of response to immunotherapy. The abundance of macrophage M0 and M2 was positively related to DCBLD2 expression, while CD8^+^ T cell infiltration had a negative correlation with DCBLD2 expression in MTAB-6134 cohort (*P* < 0.05, Figures 6A–C). Similar trends were observed in TCGA cohort (*P* < 0.05, Figures 6D–F).

### Biological Function and Pathway of DCBLD2

**FIGURE 7:**
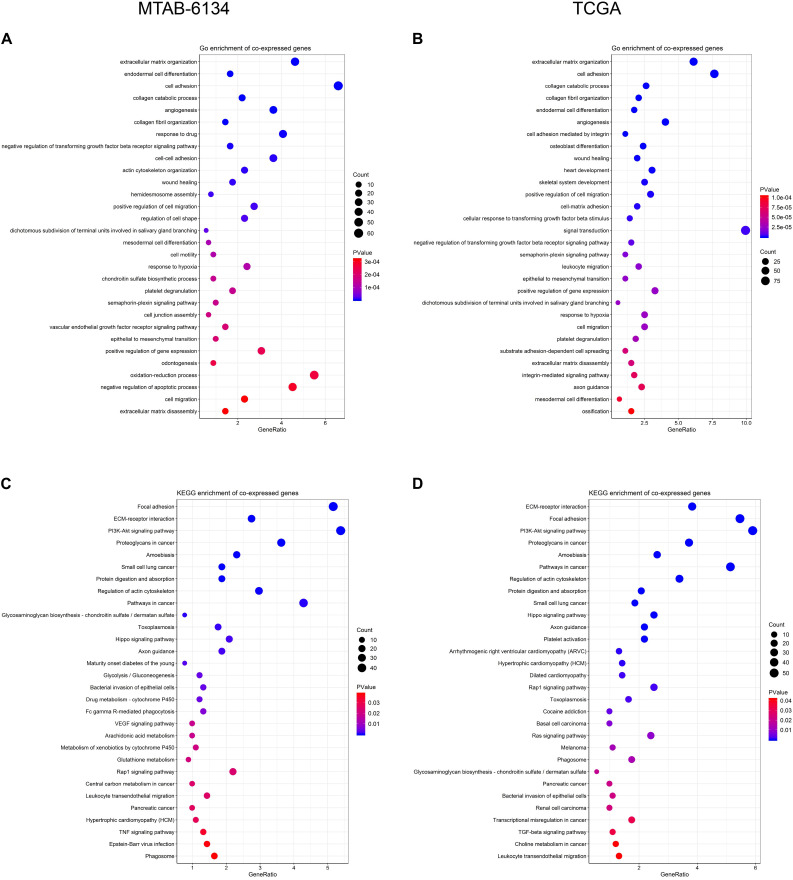
Biological function of DCBLD2. **(A,B)** Biological process analysis of top 1,000 positively co-expressed genes of DCBLD2. **(C,D)** KEGG pathway enrichment analysis of top 1,000 positively co-expressed genes of DCBLD2.

In order to preliminarily illuminate the function of DCBLD2, we performed biological process analysis and KEGG pathway enrichment analysis on top 1,000 positively co-expressed genes of DCBLD2. For biological process, DCBLD2 was found to be primarily involved in angiogenesis, cell adhesion, cell motility, and cell migration in both cohorts (Figures 7A,B). For pathway enrichment, DCBLD2 was mainly associated with PI3K-AKT signaling pathway, Hippo signaling pathway, Rap1 signaling pathway and pancreatic cancer in both cohorts (Figures 7C,D).

### Expression of DCBLD2 in Extracellular Vesicles From Human Plasma Samples

**FIGURE 8:**
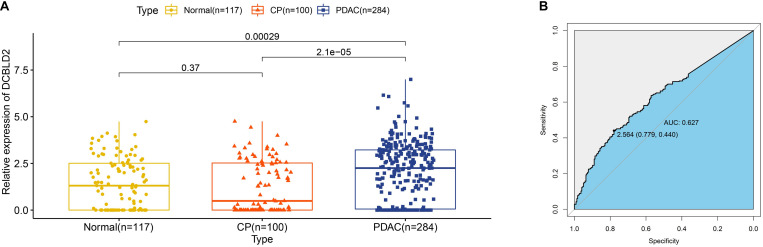
Expression of DCBLD2 in extracellular vesicles from human plasma samples. **(A)** Boxplots show the distribution of extracellular vesicular DCBLD2 expression in plasma samples from healthy donors, CP patients and PDAC patients. **(B)** ROC curve showed the diagnostic value of extracellular vesicular DCBLD2 (PDAC vs Normal + CP). The statistical significance in comparing DCBLD2 expression between the two groups was determined by Wilcoxon test.

Early diagnosis of PDAC remains challengeable, and extracellular vesicles have emerged as attractive diagnostic biomarkers for early detection of PDAC (Yee et al., 2020). Since DCBLD2 was upregulated in PDAC tissues, we wondered whether DCBLD2 was also highly expressed in extracellular vesicles from plasma samples of PDAC patients. As Figure 8A illustrated, expression of DCBLD2 in extracellular vesicles from plasma samples of PDAC patients was significantly higher than that from normal donors (*P* = 0.0029) or CP patients (*P* < 0.0001). In addition, DCBLD2 in extracellular vesicles could serve as a moderate diagnostic biomarker for PDAC as the AUC value was 0.627 (Figure 8B).

### Validation of DCBLD2 Expression in Extracellular Vesicles From Human Serum Samples

**FIGURE 9:**
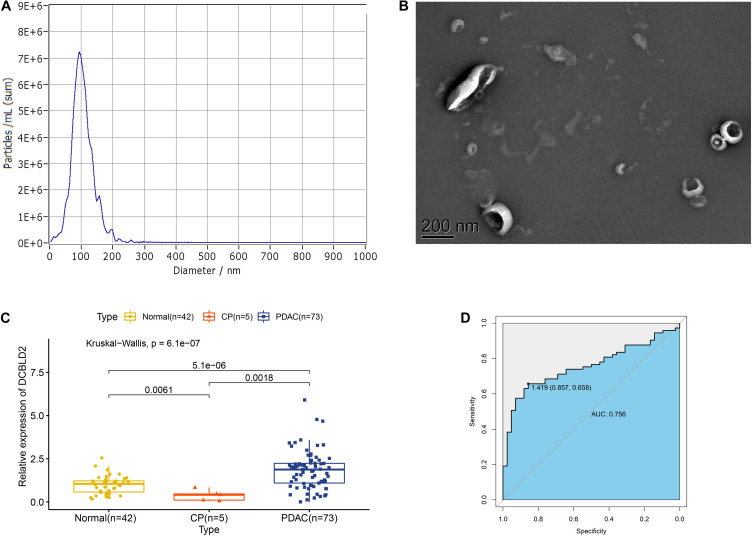
Expression of DCBLD2 in extracellular vesicles from human serum samples. **(A)** NTA analysis and **(B)** TEM analysis to assess characteristics of extracellular vesicles. **(C)** Boxplots show the distribution of extracellular vesicular DCBLD2 expression in serum samples from healthy donors, CP patients and PDAC patients. **(D)** ROC curve showed the diagnostic value of extracellular vesicular DCBLD2 (PDAC vs Normal + CP). The statistical significance in comparing DCBLD2 expression between the two groups was determined by Wilcoxon test. The statistical significance in comparing DCBLD2 expression between the three groups was investigated by Kruskal–Wallis test.

Extracellular vesicles can be isolated from both plasma and serum of whole blood, and we had proved that DCBLD2 in extracellular vesicles from plasma samples had diagnostic potential based on the public data. We next analyzed our own data to evaluate the diagnostic value of DCBLD2 in extracellular vesicles from serum samples. The results of NTA analysis and TEM demonstrated typical characteristics of isolated extracellular vesicles (Figures 9A,B). Figure 9C showed that the expression of DCBLD2 in extracellular vesicles from serum samples was markedly elevated in PDAC patients compared with healthy donors (*P* < 0.0001) or CP patients (*P* = 0.0018). Similarly, DCBLD2 in extracellular vesicles from serum samples also bore moderate diagnostic accuracy for PDAC as the AUC value was 0.756 (Figure 9D).

## Discussion

For PDAC patients, early detection is laudable and beneficial for long-term survival, but it is challenging. New strategies and better biomarkers are urgently needed to achieve early detection, especially for potentially curable PDAC (Singhi et al., 2019). In the current study, we identified a robust diagnostic and prognostic biomarker, DCBLD2, with cross-platform compatibility through bioinformatic analyses. The proposed gene exhibited satisfactory predictive performance and could be detected in extracellular vesicles from both human plasma and human serum samples. Mechanistically, DCBLD2 was correlated with immune infiltrates, cell motility and several essential oncogenic pathways.

DCBLD2 is a neuropilin-like transmembrane scaffolding receptor and participates in the regulation of receptor tyrosine kinase (RTK) signaling pathway (Nie et al., 2013; Feng et al., 2014; Li et al., 2016). Elevated expression of DCBLD2 was significantly associated with decreased OS time in various cancers including PDAC (Raman et al., 2018), colorectal cancer (Martinez-Romero et al., 2018), hypopharyngeal squamous cell carcinoma (Fukumoto et al., 2014) and melanoma (Osella-Abate et al., 2012). Apart from OS, we found that high expression of DCBLD2 was also indicative of short DFS. We previously reported that DCBLD2 was overexpressed in pancreatic cancer stem cells (Feng et al., 2020), a small population of cancer cells with an indispensable role for tumor metastasis and recurrence (Hermann et al., 2007; Simeone, 2008). These results suggested that DCBLD2 was potentially involved in the regulation of cell stemness and might serve as a promising therapeutic target for PDAC. Function analysis of DCBLD2 revealed that this gene was positively associated with several pathways participating in stemness regulation, such as PI3K-AKT signaling pathway (Qin et al., 2021; Yoon et al., 2021) and Hippo signaling pathway (Cordenonsi et al., 2011; Park et al., 2018). These findings could provide new insight into biological implications and clinical relevance of DCBLD2 in PDAC.

In addition to prognostic potential, we also attempted to characterize the diagnostic potential of DCBLD2 in tissue samples, plasma samples and serum samples. DCBLD2 expression could be a useful diagnostic biomarker to evaluate invasive properties of myxofibrosarcoma (Kikuta et al., 2017), but its diagnostic value in PDAC remained unclear. We profiled DCBLD2 expression in several cohorts containing either matched or unmatched PDAC tissues and adjacent normal tissues. Compared with normal tissues, DCBLD2 expression increased in PDAC tissues, which revealed an oncogenic role of DCBLD2. The diagnostic ability of DCBLD2 in tissues was satisfactory, as the AUC value was close to or no less than CA19-9, an established diagnostic biomarker for PDAC (Luo et al., 2020). Extracellular vesicles contain proteins, lipids and RNA from donor cells and can be an attractive source of diagnostic biomarkers for human cancers (Yu et al., 2020). More and more researches have focused on the application of extracellular vesicular protein markers in the diagnosis of human cancers (Melo et al., 2015; Yang et al., 2017). Based on the RNA-seq data from GSE133684 dataset, we found that DCBLD2 existed in extracellular vesicles from plasma samples and could serve as a moderate marker in the early diagnosis of PDAC. We also experimentally verified the diagnostic value of DCBLD2 in extracellular vesicles from serum samples. These findings highlighted the clinical utility of DCBLD2 in liquid biopsy.

Researchers recently reported two prognostic gene signatures constructed using DCBLD2 in PDAC (Raman et al., 2018; Feng et al., 2020). The prognostic accuracy of DCBLD2 was certainly inferior to that of gene models, because accumulating evidence had demonstrated that multi-gene signatures achieved higher prognostic accuracy compared with a single gene (Beane et al., 2009; Sim et al., 2017; Schmidt et al., 2018; Huang et al., 2020; Ahluwalia et al., 2021). However, we found that the diagnostic accuracy of DCBLD2 was close to or no less than that of gene models. The diagnostic AUC values of DCBLD2 were 0.800, 0.874, 0.984, and 0.818 while those of a four gene signature containing DCBLD2 (Feng et al., 2020) were 0.806, 0.881, 0.997, and 0.724 in GSE32676, GSE60979, GSE62165, and GSE71729 cohorts, respectively (Supplementary Figure 3). High diagnostic accuracy of DCBLD2 might facilitate novel development of a single-marker qPCR approach for PDAC detection with a reduced cost and widespread applicability.

To preliminarily elucidate the underlying mechanism of DCBLD2-resulted poor prognosis, we investigated the biological function and immune infiltrates associated with DCBLD2 expression. Co-expressed genes with DCBLD2 were mainly enriched in cell motility and multiple classic oncogenic pathways. We observed that the DCBLD2 expression was negatively correlated with the CD8^+^ T cells infiltration while it was positively correlated with the infiltrating level of M2 macrophage cells. Above findings further confirmed the oncogenic role of DCBLD2 and indicated that DCBLD2 had a potential impact on cell immunity. Multiple pathways related to DCBLD2 were implicated in immunological function. For instance, inhibition of PI3K-dependent phosphorylation of Akt and its transcription factor target Foxo1 resulted in defective T cell immunity (Xu et al., 2021). Hippo signaling pathway was also involved in T cell immunity (Bouchard et al., 2020) and TGF-beta signaling pathway were known to induce M2-like macrophage polarization (Gratchev, 2017). Above findings could partly explain how DCBLD2 affects the infiltration of immune cells. Therefore, this study provided the reference for clarifying the potential biological role of DCBLD2 in tumor immunology and PDAC progression.

However, this study, after all, is a retrospective study and has several limitations. First, the clinical application of DCBLD2 in PDAC management should be tested and validated in more prospective studies. Second, more *in vivo* and *in vitro* experiments are needed to verify the abovementioned bioinformatic findings, especially the biological function of DCBLD2 in PDAC tumorigenesis. Finally, we fail to adequately assess the relationship between DCBLD2 expression and clinical factors due to the lack of significant data. With the development of follow-up research, we hope to supplement them in future studies.

In conclusion, we integrated and analyzed genomic data and clinical data of multiple PDAC cohorts to demonstrate that expression value of DCBLD2 was a reliable diagnostic and prognostic factor and was significantly associated with immune and oncogenic signaling pathways in PDAC. DCBLD2 might facilitate tumor progression and bore strong diagnostic, prognostic and therapeutic value in PDAC.

Abbreviations: AUC, area under the curve; CP, chronic pancreatitis; DCBLD2, discoidin, CUB and LCCL domain-containing protein 2; DFS, disease-free survival; GEO, Gene Expression Omnibus; GEPIA, Gene Expression Profiling Interactive Analysis; KEGG, The Kyoto Encyclopedia of Genes and Genomes; K–M, Kaplan-Meier; OS, overall survival; PDAC, pancreatic ductal adenocarcinoma; ROC, receiver operating characteristic; RTK, receptor tyrosine kinase; QRT-PCR, quantitative real-time polymerase chain reaction.

## Data Availability Statement

Processed gene expression data and clinical data of TCGA cohort were obtained from the TCGA hub at UCSC Xena (https://tcga.xenahubs.net). Microarray data and clinical information of GSE21501, GSE28735, GSE57495, GSE62452, GSE71729, and GSE85916 cohorts were downloaded from the Gene Expression Omnibus (GEO) database (https://www.ncbi.nlm.nih.gov/geo/), while those of MTAB-6134 cohort were downloaded from the Arrayexpress database (https://www.ebi.ac.uk/arrayexpress/). Gene expression data of GSE15471, GSE16515, GSE32676, GSE41368, GSE55643, GSE60979, GSE62165, and GSE71989 was also downloaded from the GEO database. Normalized RNA-sequencing data and survival data of PACA-CA and PACA-AU cohorts were obtained from the International Cancer Genome Consortium (ICGC) database (https://icgc.org/).

## Ethics Statement

The studies involving human participants were reviewed and approved by the Ethics Committee of Ruijin Hospital affiliated with Shanghai Jiao Tong University. The patients/participants provided their written informed consent to participate in this study.

## Author Contributions

ZF and CP were involved in conception and design of the study and wrote the manuscript. ZF and KL participated in data analysis, discussion, and language editing. ZF collected the serum samples. YW reviewed the manuscript. All authors contributed to the article and approved the submitted version.

## Conflict of Interest

The authors declare that the research was conducted in the absence of any commercial or financial relationships that could be construed as a potential conflict of interest.

## Footnotes

1 https://www.ncbi.nlm.nih.gov/geo/

2 https://www.ebi.ac.uk/arrayexpress/

3 https://icgc.org/

4 https://tcga.xenahubs.net

5 https://www.omicshare.com/tools/Home/Soft/venn
