# Supplementary material for: Transcriptomic Profiling Identifies DCBLD2 as a Diagnostic and Prognostic Biomarker in Pancreatic Ductal Adenocarcinoma
Source: Front Mol Biosci. 2021 Mar 23;8:659168. doi: 10.3389/fmolb.2021.659168 (PMC8021715; doi:10.3389/fmolb.2021.659168)
Supplement: Supplementary file 1 [file Data_Sheet_1.PDF]

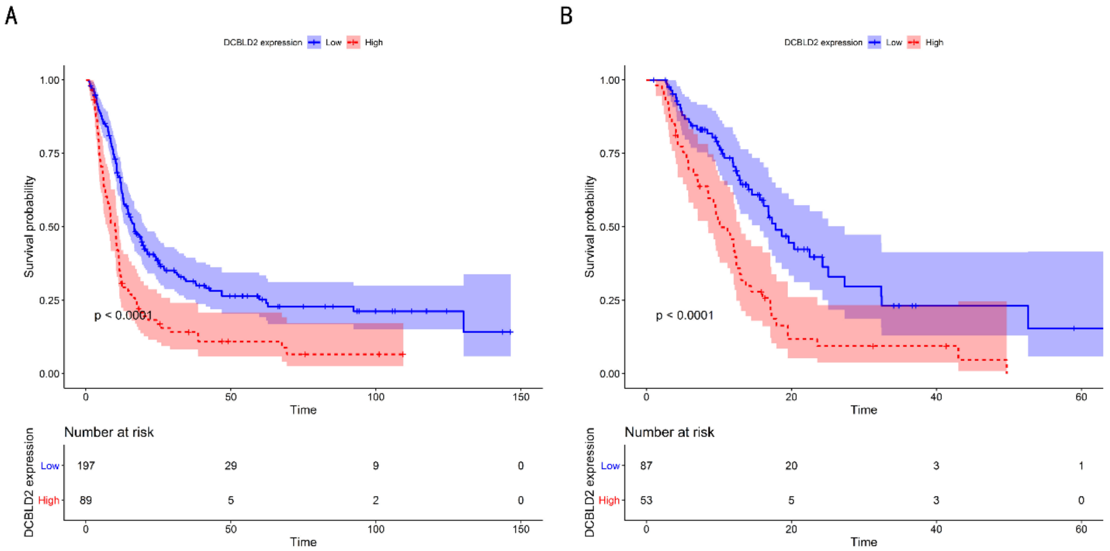

Supplementary Figure. 1 K-M survival curves showed that DCBLD2 could effectively stratify patients with different disease-free survival in MTAB-6134 cohort **(A)** and TCGA cohort **(B).** The statistical significance of differential survival was evaluated by log-rank test.

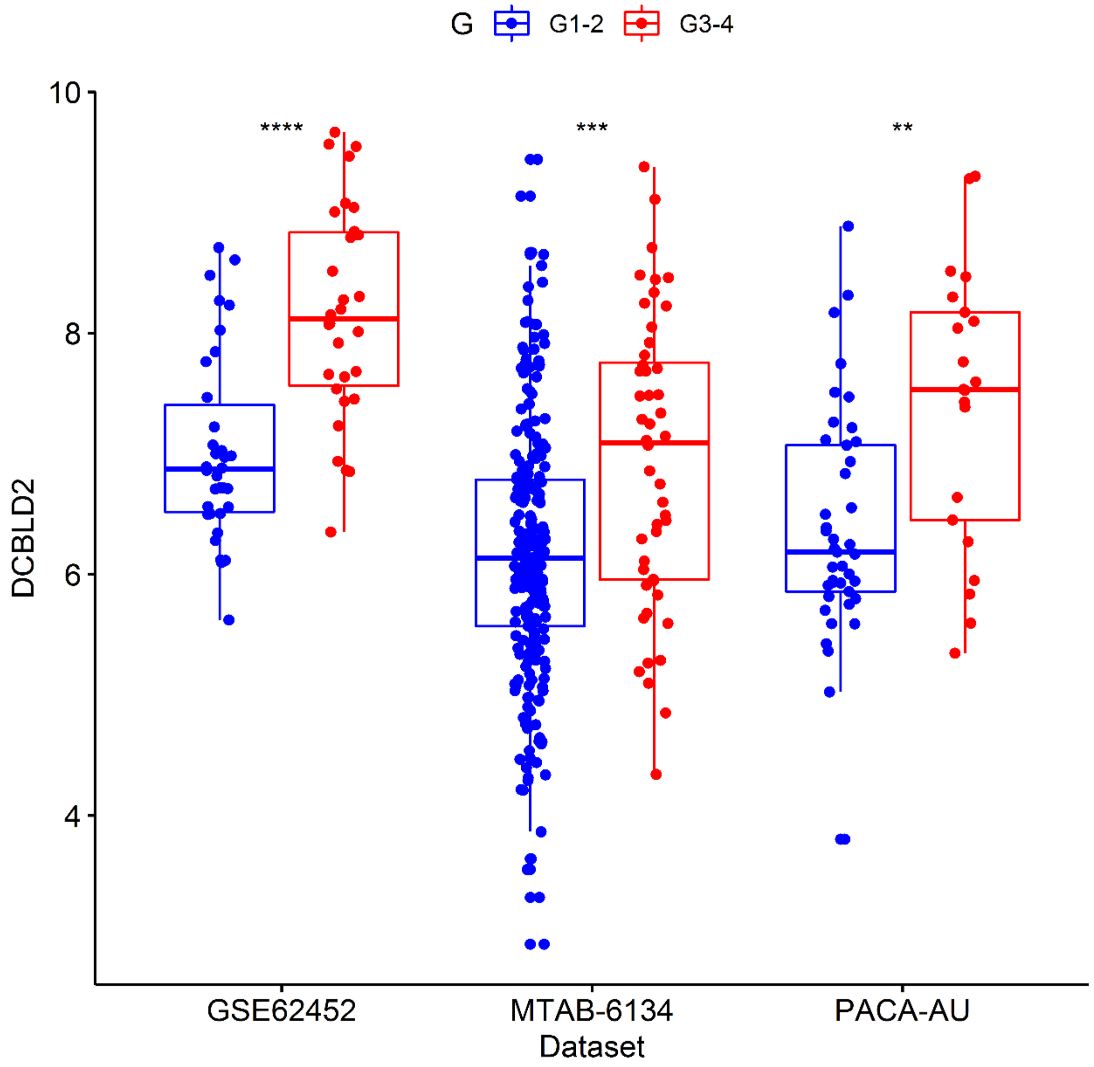

Supplementary Figure. 2 Expression of DCBLD2 in PDAC patients classified by histological grade in three independent cohorts. The statistical significance of differential expression was assessed by Wilcoxon test (*p < 0.05, **p < 0.01, ***p < 0.001, ****p < 0.0001).

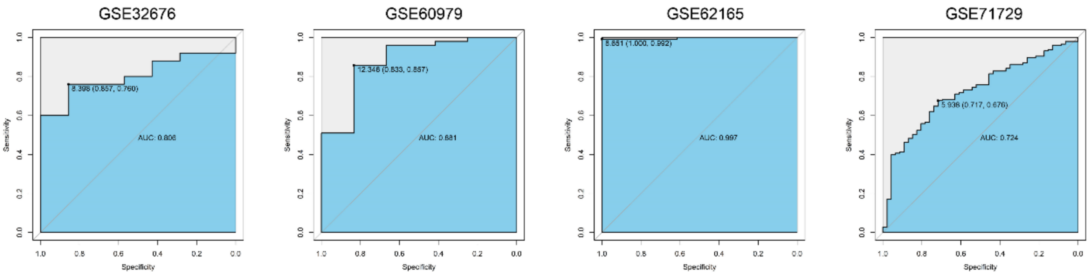

Supplementary Figure. 3 Diagnostic accuracy of the four-gene signature in four independent cohorts.
