# Supplementary material for: Transcriptomic Profiling Identifies DCBLD2 as a Diagnostic and Prognostic Biomarker in Pancreatic Ductal Adenocarcinoma
Source: Front Mol Biosci. 2021 Mar 23;8:659168. doi: 10.3389/fmolb.2021.659168 (PMC8021715; doi:10.3389/fmolb.2021.659168)
Supplement: Supplementary file 2 [file Table_1.DOCX]

Table S1. Clinicopathological characteristics of patients.

| Parameters | MTAB-6134(N=288) | PACA-AU(N=62) | TCGA(N=139) |
| --- | --- | --- | --- |
| Age at diagnosis |  |  |  |
| ≤60 | NA | 20 | 44 |
| >60 | NA | 42 | 95 |
| Gender |  |  |  |
| Male | 166 | 34 | 73 |
| Female | 122 | 28 | 66 |
| Histological grade |  |  |  |
| G1 | 110 | 1 | 17 |
| G2 | 138 | 40 | 81 |
| G3 | 48 | 20 | 40 |
| G4 | 0 | 1 | 1 |
| T stage |  |  |  |
| T1 | 12 | 1 | 4 |
| T2 | 39 | 7 | 13 |
| T3 | 237 | 52 | 119 |
| T4 | 0 | 2 | 3 |
| N stage |  |  |  |
| N0 | 72 | 19 | 34 |
| N1 | 216 | 43 | 105 |
| Residual tumor |  |  |  |
| R0 | 235 | NA | 77 |
| R1 | 49 | NA | 47 |
| R2 | 0 | NA | 5 |
| NA | 4 | NA | 10 |
